# Dietary acid load significantly predicts 10-years survival in patients underwent coronary artery bypass grafting (CABG) surgery

**DOI:** 10.1371/journal.pone.0223830

**Published:** 2019-10-17

**Authors:** Mahdieh Abbasalizad Farhangi, Mahdi Vajdi, Mahdi Najafi

**Affiliations:** 1 Nutrition Research Center, Tabriz University of Medical Sciences, Tabriz, Iran; 2 Drug Applied Research Center, Tabriz University of Medical Sciences, Tabriz, Iran; 3 Student Research Committee, Department of Community Nutrition, Tabriz University of Medical Sciences, Tabriz, Iran; 4 Department of Research, Tehran Heart Center, Tehran University of Medical Sciences, Tehran, Iran; 5 Faculty Research Associate, Western University, London, ON, Canada; 6 Cardiac Outcome Research and Education (CORE), Universal Scientific Education and Research Network (USERN), Tehran, Iran; University Magna Graecia of Catanzaro, ITALY

## Abstract

**Backgrounds:**

Numerous studies have revealed the role of dietary acid load as a potential risk factor for cardiovascular events and blood pressure. However, its role in predicting the mortality rate in patients underwent coronary artery bypass grafting surgery (CABG) has not been reported. In the current study we aimed to evaluate the relationship of dietary acid load and cardio-metabolic risk factors with ten year survival among patients underwent CABG.

**Methods:**

The current prospective cohort study comprises 454 patients underwent CABG. Anthropometric, clinical and biochemical measurements were performed. Dietary acid load was calculated as either potential renal acid load (PRAL) or net endogenous acid production (NEPA) using the data obtained from a semi-quantitative food frequency questionnaire (FFQ). Survival analysis was performed using Kaplan-Meier method followed by log-rank test. The association between all-cause mortality and study parameters was performed with Cox-proportional hazard model.

**Results:**

Patients in the higher PRAL and NEAP quartiles had lower BMI and lower ejection fraction rate (P <0.05). Moreover, lower hematocrit values were observed in patients of higher PRAL quartiles. Higher PRAL scores were associated with higher mortality rate and reduced survival days (adjusted hazard ratio: 1.023 (1.00–1.04; P-value = 0.01). However, there was no relationship between NEAP and survival.

**Conclusions:**

We revealed that high PRAL scores are positive predictors of 10-year mortality in patients underwent CABG. The results of our study suggest that maintaining an adequate acid-base balance can contribute to longevity by reducing the risk of mortality.

## Introduction

Cardiovascular disease (CVD), as one of the most important causes of death and disability in the world, is responsible for more than 12% of the universal disease burden [1,2]. In 2015, an estimated 17.7 million people died from CVDs, out of them around 6.7 and 7.4 million were due to stroke and coronary heart disease (CHD) respectively [3]. In recent decades, CVD mortality has decreased in developed countries but in middle-and low-income countries, CVD is still the main cause of death [1]. In Iran, also, CVDs including stroke, vascular diseases and coronary artery disease (CAD) are the main components of non-communicable disease (NCD) burden [4]. Similar to most of the middle -and low -income countries, in Iran, 79% of deaths are related to chronic diseases and 50% of all deaths per year are related to CVD [4,5]. It has been estimated that the burden of cardiovascular disease in Iran will increase over 2005 to 2025, mostly because of the increase aging population and demographic and epidemiologic transitions [6]. Coronary artery bypass grafting (CABG) is often chosen when atherosclerosis of one or more of coronary arteries have a 50 to 99 percent stenosis [7]. Well-known risk factors of CVD are poor eating habits and unhealthy diet, sedentary lifestyle, hypertension, diabetes mellitus, smoking, dyslipidemia and family history of premature coronary artery disease [8]. Diet plays a main role among the many determined risk factors for CVD. The modification of dietary habits with increase physical activity could considerably reduce mortality and increase life expectancy [9,10]. Dietary intake can substantially impact the body’s acid-base status [11]. Acid/base stability in body is one of the vital factors that impact the human health [12]. Increasing fruit and vegetable intake and lowering protein, and potassium salts or magnesium supplements have been shown to normalize the body's acid-base balance [13]. Imbalance in the intake of alkalizing or acidifying foods is attributed to lower serum bicarbonate and urine pH [14]. Several studies have been focused on the impact of diet-induced acid load on cardiometabolic risk factors [13,15]. A recent study reported that excess acidity and alkalinity of diet were linked with a higher risk of mortality in Swedish adults [16]. Moreover, numerous studies have shown that high dietary acid load has been related to an undesirable profile of cardiometabolic risk factors including hypertension [17,18], insulin resistance [19], hypertriglyceridemia [17], type 2 diabetes [20,21], high LDL cholesterol [17] and central obesity [22]. A diet with low alkaline foods, such as fruits and vegetables, legumes and high acidogenic foods, such as fish, cheese and meat, eggs, rice, dairy products and cereals can increase endogenous acid production [13]. Dietary acid load has been indicated by net endogenous acid production (NEAP) and potential renal acid load (PRAL). NEAP, estimates the diet acidity according to ingested protein and potassium and PRAL estimation includes dietary calcium, phosphorus, magnesium, potassium and protein [12,23]. Higher dietary acid load values and positive PRAL values reflect acid-forming potential while lower DAL scores and negative scores of PRAL indicate base-forming potential [24]. To best of our knowledge the potential predictability of dietary acid load for long-term survival in patients underwent CABG surgery has not been evaluated yet. Therefore in the current study we aimed to evaluate the relationship of dietary acid load, represented by both NEAP and PRAL scores, with cardiometabolic risk factors and 10 year survival among patients underwent CABG.

## Method and materials

### Subjects

This cross-sectional study was carried out among 454 CAD patients aged between 35 to 80 years old candidates for isolated CABG with cardiopulmonary bypass and were enrolled for Tehran Heart Center Coronary Outcome Measurement (THC-COM) study (Tehran heart center, Iran) [25–27]. The study was performed between May-September 2006. The participants were followed up to 10 years. Participants in this study were patients admitted to the cardiothoracic ward for CABG surgery at a large Heart Center in this time period (Tehran heart center, Iran). Exclusion criteria were attendant repair or replacement of heart valve, ventricular aneurism resection or any surgeries other than CABG. The method of sample size calculation has been described before; concisely, The formula for comparing two proportions: n = [(Zα/2 +Zβ)2 × {(p1 (1-p1) + (p2(1-p2))}] / (p1−p2)^2^ was used considering p1 and p2 as the proportions of the women and men with low quality Mediterranean regimen (0.3 and 0.25), power of 80% and α of 0.05. consequently with supposing 20% lost to follow-up a total sample size of 450-was enrolled [25–27]. Written informed agreement was provided from each patient. Clinical assessment and pre-operative cardiac status was measured by several variables such as: number of diseased vessels, New York Heart Association (NYHA) functional class, left ventricular ejection- fraction and the European system for cardiac operative risk evaluation (EuroSCORE) [28]. Anthropometric variables including body mass index (BMI) was calculated and height and weight were measured. Height was measured in a standing position without shoes. Weight was measured using digital scale while subjects wearing light clothes and without shoes [29].

### Dietary assessment methods and dietary acid load calculation

Dietary acid load was calculated based on a 138-item semi-quantitative food frequency questionnaire (FFQ) consisting of a list of foods with standard serving sizes usually consumed by Iranians and was adopted and validated for use in Iran [30]. All questionnaires were administered by a trained researcher who was blind for the data collection. The interviewer asked participants to report frequency of the consumed food items during the previous year on the number of times per day, per week, per month or per year. The reported frequency for each food item was then converted to a daily intake. Portion sizes of consumed foods were converted to grams by using household measures [31]. Dietary acid load was evaluated by two formulas: NEAP (Pro: K) and PRAL and quartile of the scores were used for statistical analysis: NEAP was calculated as dietary protein (g/day) divided by dietary potassium (mg/day) [12] and PRAL was calculated using the following formula:

$PRAL\;\left(\frac{mEq}{day}\right)=0.4888\times dietary\;protein\;\left(\frac{g}{day}\right)+0.0366\times dietary\;phosphorus\;\left(\frac{mg}{day}\right)–0.0205\times dietary\;potassium\;\left(\frac{mg}{day}\right)–0.0125\times calcium\;\left(\frac{mg}{day}\right)–0.0263\;magnesium\;(mg/day)$ [13]. Higher scores of NEAP and PRAL indicate a more acidic dietary acid-base load [32].

### Statistical analysis

Analysis of data was performed by SPSS software (statistical package for social analysis, version 23, SPSS Inc., Chicago, IL, USA).The normality of data was tested by Kolmogorov-Smirnov test and all parameters were normally distributed. Data are presented as number (N), or percent (%) for categorical variables and mean and standard deviation (SD) for continuous variables. We analyzed the study participants’ characteristics according to PRAL and NEAP quartiles, using one way analysis of variance (ANOVA) to compare continuous variables, χ2 tests for categorical variables followed by post hoc analyses with the Bonferroni method. Survival analysis was performed using Kaplan-Meier method followed by log rank test. P values less than 0.05 were considered statistically significant.

## Result

**Fig 1:**
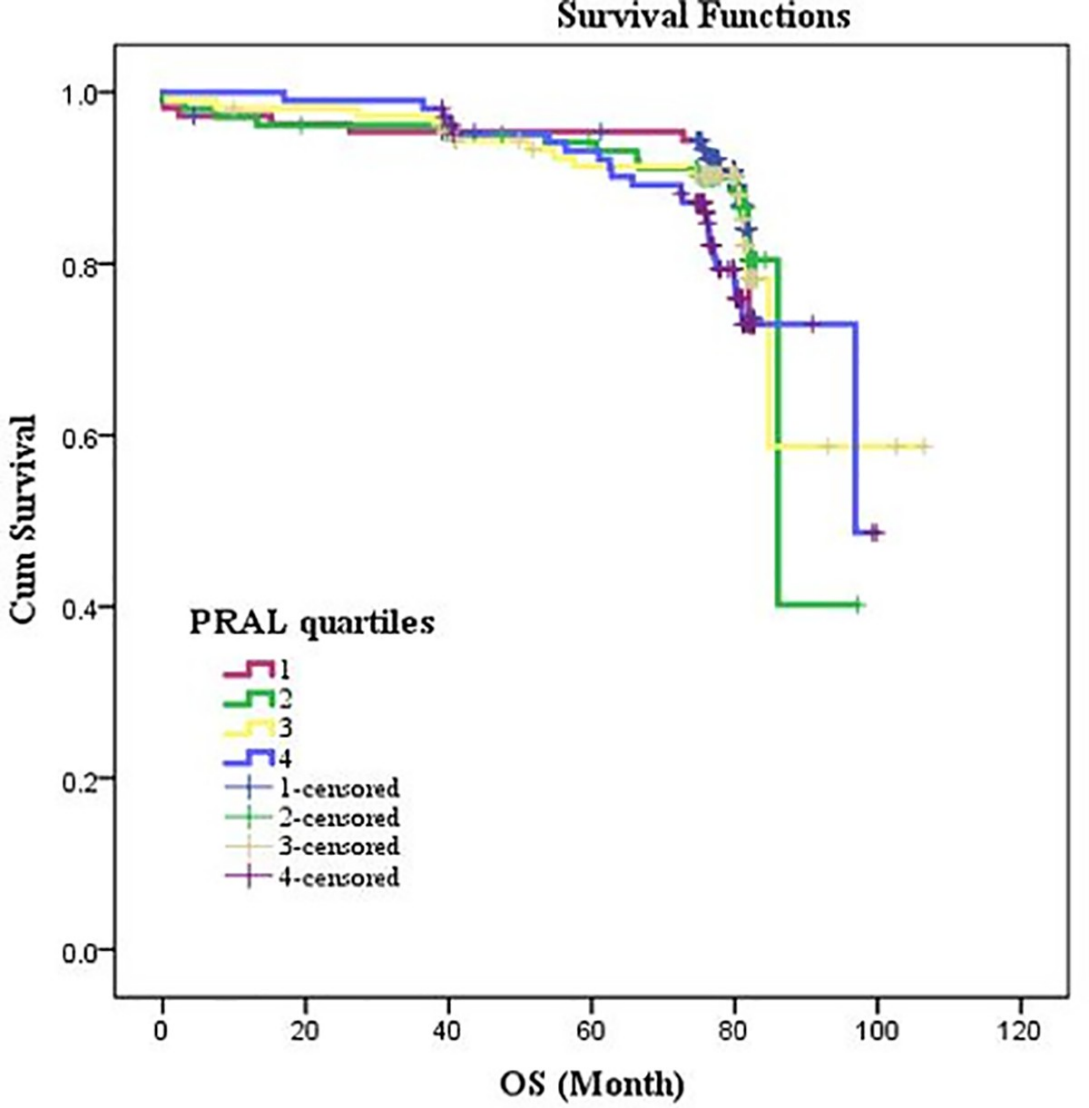
PRAL and estimated operation to survival (OS) after 10 years in patients underwent CABG.

**Fig 2:**
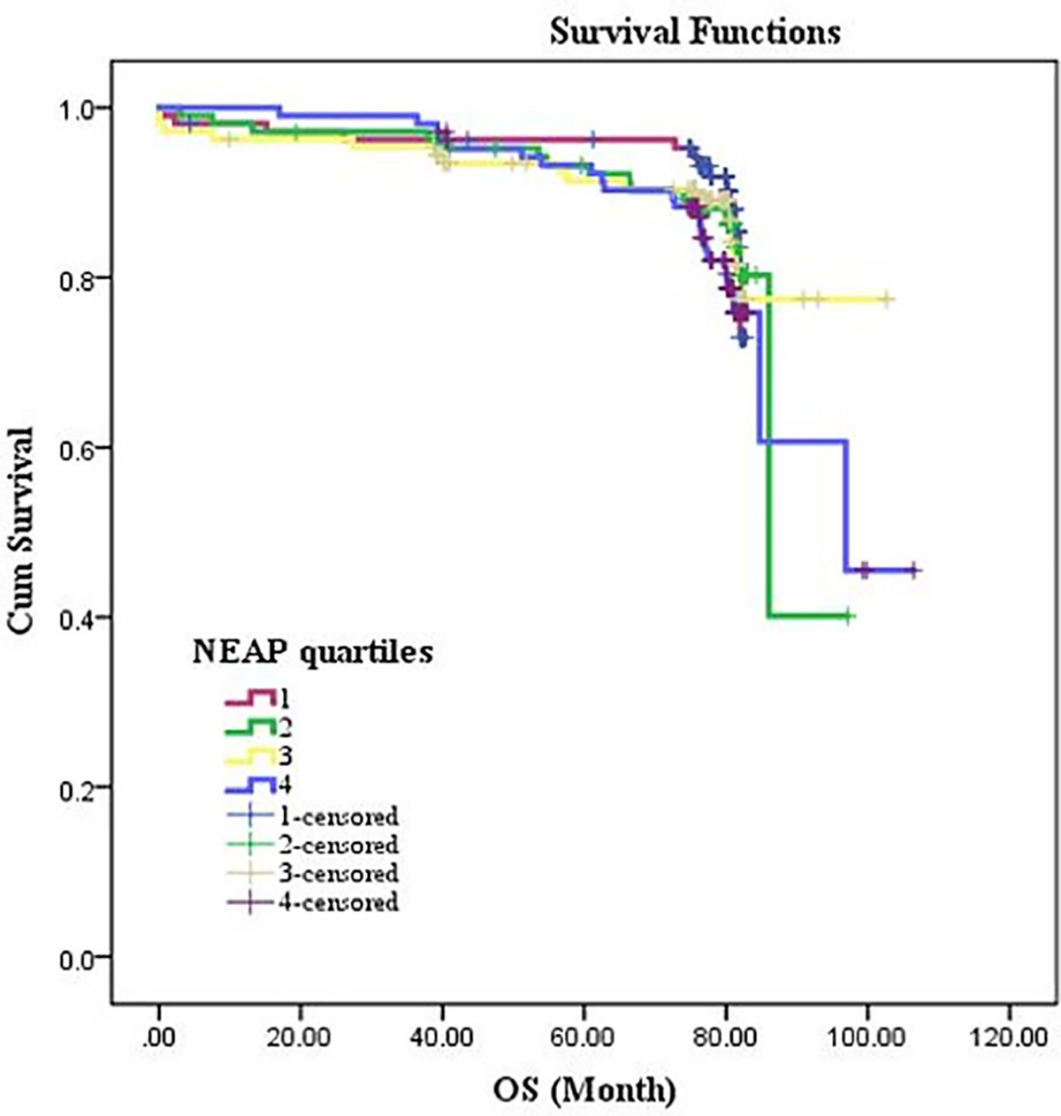
NEAP and estimated operation to survival (OS) after 10 years in patients underwent CABG.

Table 1 presents the general demographic and clinical parameters among patients according to PRAL quartiles among patients. Patients in the top quartile of PRAL, had significantly lower BMI and lower ejection-fraction rate compared with lower quartiles (P = 0.02 and P< 0.003 respectively). Also, patients in 1^st^ quartiles of PRAL had significantly higher HCT concentrations compared with other quartiles (P < 0.03; Table 2). Tables 3 and 4 demonstrate the demographic and biochemical variables according to NEAP quartiles. As shown, patients in the top quartile of NEAP, had significantly lower BMI compared with patients in lower quartiles (P = 0.003). Gender distribution was also in favor of men in top quartiles of NEAP. Patients in the highest NEAP quartile had significantly lower rate of EF (P< 0.03). There were no significant differences in other parameters across quartiles of PRAL or NEAP. Table 5 shows the patients in the top quartile of DAL (PRAL and NEAP), have higher dietary intake of energy, macronutrients and several micronutrients among all participants. Patients in the higher quartiles of dietary acid load, had significantly higher energy, protein, carbohydrate, fat, saturated fatty acids, poly and monounsaturated fatty acids, cholesterol, Na and phosphorus intake while lower consumption of fiber, vitamin A, E, K, C, calcium and potassium. The survival rate of patients underwent CABG according to PRAL and NEAP scores are presented in Figs 1 and 2. Higher PRAL scores were associated with increased total mortality in patients underwent CABG (adjusted HR for age, gender and BMI: 1.023 (1.00–1.04; P-value = 0.01; Table 6). The Log Rank test (Table 7) revealed that there is significant difference in the survival rate in highest versus lowest quartiles of PRAL (P = 0.012), while no significant difference in the survival rate in highest versus lowest quartiles of NEAP was observed (P = 0.20).

**Table 1:** General demographic and anthropometric parameters in participants according to PRAL quartiles.

Quintiles of PRAL score					
Variable	1^st^ quartile	2^nd^ quartile	3^rd^ quartile	4^th^ quartile	P value
	N = 113	N = 114	N = 114	N = 113	
**Age (y)**	59.07±8.92	59.96±8.19	58.68±8.89	58.30±9.86	0.54
**Gender male [n (%)]**	81 (71.7)	77(67.5)	87(76.3)	88(77.9)	0.17
**BMI (kg/m**^**2**^**)**	28.34 ± 4.57	26.94 ± 3.65	27.57±3.80	26.89±3.76	0.02*
**Diabetic [n (%)]**	48 (42.5)	47(41.2)	52(45.6)	45(39.8)	0.83
**High education level [n (%)]**	16 (14.2)	17(15.9)	17(15.3)	16(14.4)	0.57
**Smokers [n (%)]**	38 (33.6)	37(32.7)	42(36.8)	42(37.2)	0.86
**Hyperlipidemia [n (%)]**	88 (77.9)	80(70.2)	74(64.9)	81(71.7)	0.19
**EF (mean)**	48.05 ± 9.86	50.92 ± 10.33	48.99 ±11.0	45.85±9.41	0.003*
**Hypertension [n (%)]**	54(47.8)	57(50.4)	49(43.0)	57(50.4)	0.63
**Opium**	18(15.9)	15(13.3)	18(15.8)	20(17.9)	0.82
**Alcohol**	16(15.2)	17(16.2)	14(13.6)	11(10.6)	0.66
**MI [n (%)]**	60(53.1)	54(47.8)	53(47.3)	60(53.1)	0.70

BMI, body mass index; MI, myocardial Infarction; P value for discrete variables based on Chi-Square Test and for continuous variables based on ANOVA. Discrete and continuous variables data are presented as number (percent) and mean (SD). High educational attainment was defined as educational level more than 12 years.

* Indicates statistically significant values as P<0.05

**Table 2:** Biochemical variables of patients underwent CABG according to PRAL quartiles.

Quintiles of PRAL score					
Variable	1^st^ quartile	2^nd^ quartile	3^rd^ quartile	4^th^ quartile	P value
	N = 113	N = 112	N = 114	N = 113	
**HbA**_**1**_**C (%)**	6.25±1.64	5.95±1.60	6.15±1.41	6.06±2.24	0.64
**TC (mg/dl)**	165.29±45.86	158.20±49.64	155.58±40.77	164.71±46.19	0.29
**TG (mg/dl)**	175.58±79.81	176.92±92.18	161.95±71.09	185.39±112.62	0.27
**LDL (mg/dl)**	88.86±36.92	83.96±45.92	84.36±36.45	90.83±39.84	0.48
**HDL (mg/dl)**	40.05±8.67	40.92±10.61	40.29±7.43	40.74±8.63	0.87
**HCT (%)**	43.16±9.87	40.93±3.93	42.26±4.36	42.78±4.18	0.03*
**Albumin (g/dL)**	4.65±0.32	4.62±0.29	4.66±0.32	4.69±0.38	0.43
**Creatinine (mg/dL)**	1.28±0.21	1.30±0.29	1.32±0.31	1.29±0.26	0.80
**BUN (mg/dL)**	39.93±12.06	41.14±12.42	41.20±13.49	39.35±10.58	0.59
**LP (a) (mg/dL)**	30.39±24.19	32.93±29.57	33.41±25.71	33.26±25.82	0.80
**CRP (mg/dL)**	7.34±8.23	6.52±4.52	6.47±3.94	6.66±2.95	0.58
**Number of diseased vessels**	2.72 ±0.49	2.79±0.46	2.75±0.50	2.65±0.59	0.21

Hb, hemoglobin; TC, total cholesterol; TG, triglyceride; LDL, low density lipoprotein cholesterol; HDL, high density lipoprotein cholesterol; HCT, hematocrit; BUN, blood urea nitrogen; CRP, C-reactive protein.

* Indicates statistically significant values as P<0.05.

**Table 3:** Biochemical variables of patients underwent CABG according to NEAP quartiles.

Quartiles of NEAP score					
Variable	1^st^ quartile	2^nd^ quartile	3^rd^ quartile	4^th^ quartile	P value
	N = 113	N = 112	N = 113	N = 113	
**Age (y)**	59.09±8.57	59.58±8.50	58.46±9.45	58.88±9.41	0.82
**Gender male [n (%)]**	70.8))80	80 (70.2)	79(69.3)	94(83.2)	0.04*
**BMI (kg/m**^**2**^**)**	28.11±4.71	26.92±3.44	28.16±3.83	26.55±3.67	0.003*
**Diabetic [n (%)]**	49(43.4)	43(37.7)	52(45.6)	48(42.5)	0.67
**High education level [n (%)]**	13(11.7)	23(21.1)	15(13.1)	15(13.1)	0.10
**Smokers [n (%)]**	32(28.3)	45(39.8)	40(35.1)	42(37.2)	0.30
**Hyperlipidemia [n (%)]**	89 (78.8)	75 (65.8)	78 (68.4)	81(71.7)	0.15
**EF (mean)**	48.64 ± 9.80	50.73±10.70	47.52±10.31	46.95±10.11	0.03*
**Hypertension [n (%)]**	55 (48.7)	51(45.1)	61(53.5)	50(44.2)	0.49
**Opium**	14 (12.4)	23(20.4)	14(12.3)	20(17.9)	0.24
**Alcohol**	13 (12.6)	19 (18.1)	16(15.1)	10(9.7)	0.34
**MI [n (%)]**	53 (46.9)	61(54.0)	60(53.6)	53(46.9)	0.54

BMI, body mass index; MI, myocardial Infarction; P value for discrete variables based on Chi-Square Test and for continuous variables based on ANOVA. Discrete and continuous variables data are presented as number (percent) and mean (SD). High educational attainment was defined as educational level more than 12 years.

* Indicates statistically significant values as P<0.05

**Table 4:** Biochemical variables of patients underwent CABG according to NEAP quartiles.

Quartiles of NEAP score					
Variable	1^st^ quartile	2^nd^ quartile	3^rd^ quartile	4^th^ quartile	P value
	N = 113	N = 112	N = 113	N = 113	
**HbA**_**1**_**C (%)**	6.22±1.64	5.91±1.55	6.12±1.47	6.16±2.23	0.57
**TC (mg/dl)**	164.30±47.59	154.31±41.87	165.13±49.65	160.01±43.21	0.26
**TG (mg/dl)**	178.93±85.75	160.68±75.06	182.93±89.39	177.12±107.63	0.26
**LDL (mg/dl)**	89.02±37.93	81.02±36.51	89.02±45.45	88.95±39.03	0.33
**HDL (mg/dl)**	39.68±8.60	41.88±9.67	40.24±9.20	40.20±7.92	0.26
**HCT (%)**	43.08±9.83	41.37±4.18	41.98±4.19	42.70±4.35	0.16
**Albumin (g/dL)**	4.67±0.32	4.61±0.28	4.67±0.32	4.68±0.39	0.36
**Creatinine (mg/dL)**	1.27±0.22	1.31±0.28	1.30±0.32	1.30±0.26	0.66
**BUN (mg/dL)**	40.55±12.01	39.04±11.29	41.65±14.07	40.42±11.12	0.45
**LP (a) (mg/dL)**	30.40±24.13	32.50±28.91	35.92±27.28	31.16±24.65	0.41
**CRP (mg/dL)**	7.06±7.74	6.52±4.57	7.17±5.21	6.26±2.33	0.52
**Number of diseased vessels**	2.73±0.48	2.76±0.48	2.76±0.52	2.56±0.56	0.28

Hb, hemoglobin; TC, total cholesterol; TG, triglyceride; LDL, low density lipoprotein cholesterol; HDL, high density lipoprotein cholesterol; HCT, hematocrit; BUN, blood urea nitrogen; CRP, C-reactive protein.

**Table 5:** The comparison of dietary energy, macronutrients and several micronutrient intake according to dietary acid load quartiles.

Quintiles of DAL score						
Variable		1^st^ quintile	2^nd^ quintile	3^rd^ quintile	4^th^ quintile	P value
		N = 113	N = 114	N = 114	N = 113	
**Energy(Kcal)**	PRAL	2773.73±1194.58	2552.09±903.41	2600.18±908.53	3368.87±1674.58	**<0.001**
	NEAP	2544.69±906.27	2665.63±952.22	2961.15±1379.06	3119.21±1565.21	**<0.001**
**Fiber(g)**	PRAL	43.31±17.43	35.34±15.46	32.92±13.21	41.13±28.78	**<0.001**
	NEAP	40.18±15.22	38.03±17.33	38.32±21.81	36.10±24.40	0.50
**Protein(g)**	PRAL	96.34±34.36	91.44±28.26	95.89±31.72	126.30±63.59	**<0.001**
	NEAP	87.26±27.32	94.99±27.99	104.68±39.85	122.93±62.99	**<0.001**
**Carbohydrate(g)**	PRAL	437.62±212.63	389.43±157.86	391.60±153.34	511.51±293.57	**<0.001**
	NEAP	398.06±150.12	410.35±173.96	457.39±253.35	463.59±262.77	**0.04**
**Fat (g)**	PRAL	81.11±39.58	77.18±30.51	78.23±30.57	96.33±56.67	**<0.001**
	NEAP	76.67±37.29	79.24±29.72	85.78±40.76	91.08±53.05	**0.03**
**SFA (mg)**	PRAL	31.23±17.30	29.28±11.73	29.79±11.61	36.99±21.17	**<0.001**
	NEAP	29.83±16.64	29.90±11.35	32.91±15.31	34.62±20.01	**0.06**
**MUFA (mg)**	PRAL	30.10±14.42	28.95±11.96	30.00±12.54	37.87±23.15	**<0.001**
	NEAP	28.67±13.71	30.03±11.94	33.08±16.33	35.11±21.72	**0.01**
**PUFA (mg)**	PRAL	18.25±8.53	18.27±9.65	17.51±7.91	23.88±15.07	**<0.001**
	NEAP	17.06±7.70	19.44±9.97	20.79±11.66	20.58±13.33	**0.03**
**Cholesterol (mg)**	PRAL	246.53±148.48	237.76±110.17	264.08±156.75	330.73±224.07	**<0.001**
	NEAP	236.02±153.14	240.28±124.46	266.05±119.21	336.71±233.82	**<0.001**
**Vitamin A (RAE/ d)**	PRAL	1787.42±1020.63	1377.01±661.97	1236.99±677.54	1392.83±1566.84	**<0.001**
	NEAP	1679.19±1027.22	1448.09±712.95	1265.56±633.92	1400.52±1583.22	**0.03**
**Vitamin E (mg)**	PRAL	11.26±4.15	9.65±3.01	9.62±3.61	10.64±5.46	**<0.001**
	NEAP	10.59±3.99	10.21±3.46	10.35±4.18	10.02±5.05	0.77
**Vitamin K (μg/d)**	PRAL	708.39±397.64	558.37±284.23	519.60±357.33	436.60±261.93	**<0.001**
	NEAP	635.93±276.88	636.30±451.06	533.38±299.16	416.54±266.16	**<0.001**
**Vitamin C (mg)**	PRAL	291.17±118.04	211.38±73.13	188.90±71.34	171.37±100.56	**<0.001**
	NEAP	268.79±112.06	220.41±76.53	203.78±95.87	169.63±100.82	**<0.001**
**Calcium (mg)**	PRAL	1426±47±497.49	1240.46±373.52	1195.56±386.63	1270.60±517.50	**<0.001**
	NEAP	1327.66±445.05	1277.14±378.88	1300.57±489.12	1226.46±497.57	0.38
**Na (mg)**	PRAL	2815.03±1943.44	2596.68±1145.84	2556.08±1021.51	3386.47±1875.93	**<0.001**
	NEAP	2500.454±1019.54	2672.72±1227.45	3106.51±2030.62	3069.03±1768.61	**<0.001**
**Phosphorus (mg)**	PRAL	1863.56±635.74	1685.59±564.03	1717.87±579.20	2162.86±1084.35	**<0.001**
	NEAP	1726.62±568.62	1781,85±583.31	1921.53±790.09	1997.22±1019.81	**0.03**
**Potassium (mg)**	PRAL	7066.19±2119.99	5365.40±1513.97	4889.81±1567.69	5289.56±2593.27	**<0.001**
	NEAP	6573.45±2097.22	5627.11±1655.34	5363.11±2059.15	5040.79±2465.10	**<0.001**

**Table 6:** The adjusted Cox Regression model for the relationship between dietary acid load and ten-year survival in patients underwent CABG.

**Variable**	**Crude HR (95% CI)**	**P value** ^**&**^	**Adjusted HR (95% CI)**	**P value**
PRAL	1.02 (1.00–1.04)	0.02	1.02 (1.00–1.04)	0.01*
NEAP	0.96 (0.91–1.02)	0.21	0.95 (0.90–1.01)	0.12

^&^ The P value and confidence interval (CI) was estimated using cox regression model adjusting for the confounding effects of age, gender, BMI.

* Indicates statistically significant values as P < 0.05.

**Table 7:** Test of equality of survival distribution for the different levels of PRAL quartiles and NEAP quartiles.

Log Rank (Mantel-Cox)	Chi-Square	df	P value*
PRAL	2.00	1	**0.012**
NEAP	1.61	1	0.20

* Indicates statistically significant values as P < 0.05

## Discussion

To our knowledge, this is the first and the biggest study to survey the association of dietary acid load with 10-years survival in Iranian patients underwent CABG surgery. We evaluated dietary acid load using two formulas: NEAP and PRAL scores. We demonstrated positive association between higher PRAL scores and mortality rate and patients in the top quartile of PRAL and NEAP, had significantly lower BMI and EF compared with patients in lower quartiles. Also, patients in higher quartiles of PRAL had significantly lower HCT values. High phosphorus and protein and low magnesium, potassium and calcium intake can affect body's acid-base balance. In the previous part of the project, we revealed that high dietary total antioxidant capacity (TAC) could be considered as a potent protective tool against cardio-metabolic risk factors in the same population especially in male subjects [33]. In the current study we examined the role of DAL in prediction of survival and our results revealed that high PRAL scores could be considered as a positive predictor of mortality in patients underwent CABG. Several studies suggested the increased risk of CVD in higher PRAL score possibly due to increased insulin resistance, blood pressure, adiposity and incidence of diabetes or hypertension [17–21,34,35]. Former studies have shown, individuals with higher dietary acid load have a tendency to unhealthy lifestyle patterns, including higher BMI, sedentary life-style and great interest to western diet patterns [18,21]. Akter S et al [36] found that higher PRAL value was associated with an increased risk of CVD mortality, though no association with cancer mortality was reported. Accordingly, XuH et al, found a higher risk of CVD mortality in higher PRAL score [16]. A study on Korean people found that biomarkers of higher metabolic acid load were related to CVD mortality [37]. In the current study, BMI tended to decline with quartiles of NEAP and PRAL. In the study by Najafi M et al a significant HR was observed for BMI in predicting overall mortality [38]. The phenomenon obesity-mortality paradox which is generally accepted in short term outcome studies is described by better result in patients with higher BMI compared to the others. But, there is no consensus in long term examinations as Del Prete et al reported that long term survival was not significantly different between non-obese and obese patients after making adjustment model [39]. A meta-analysis by Oreopoulos A et al [40], found that obesity and overweight possibly have good effect on mortality after angioplasty and vascular bypass. In study of 10268 patients who underwent isolated CABG, morbid obesity associated with late mortality while underweight related to early mortality [41]. Birkmeyer NJ et al [42] found that obesity among patients undergoing CABG was not related with increased mortality and obesity was related to reduce risk of bleeding after surgery. Also, patients in the top quartile of DAL had significantly lower rate of EF compared with patients in lower quartiles. In our previous study we found that lower ejection fraction rate related with decrease survival in patients underwent CABG surgery [43]. Similar to our results, Najafi M et al demonstrated that lower EF is a weighty forecaster for higher rates of all-cause mortality of patients undergoing CABG surgery and patients with a low EF had a less survival than did patients with a normal EF [38]. In our study, patients in lowest quartiles of PRAL had significantly higher HCT concentrations compared with other quartile. Reduced hemoglobin and hematocrit concentrations have been reported to be associated with poor prognosis and functional impairment in Patients with advanced heart failure [44]. Low hemoglobin and hematocrit is associated with increased mortality in patients with cardiovascular disease [45]. Reduced preoperative HCT concentration has also been shown to be associated with increased stroke rate in the 30-day period following CABG [46]. The possible underlying mechanisms of the inverse association between dietary acid load and HCT is possibly because of increased calcium absorption and consequent reduced iron absorption in a competition manner [47] or even reduced dietary vitamin C intake as one of the most important iron absorption facilitators in higher PRAL categories in the current study. In current study PRAL had significantly inverse association with dietary vitamins C and E intakes. Antioxidants such as vitamin C and E may prevent atherosclerotic plaque development by modifying platelet activity and vascular reactivity, inhibiting LDL-cholesterol oxidation and reducing thrombotic potential [48,49].

In a pooled study, vitamin C supplement was significantly related with a 25% reduction in CHD risk [50].

However, in the long-term randomized clinical trial of male physicians, neither vitamin E nor C supplementation reduced the risk of major cardiovascular events [51]. In study by Rimm EB et al it has been demonstrated that risk of CAD in participants of top quintile of vitamin E consumption was 41% lower than patients in low quintile [52]. The current study also has several limitations. Firstly, the self-reported dietary information gathered by FFQ could address a potential recall bias. However, the validity and reliability of the FFQ had been confirmed before. Secondly, we did not directly measure urine or plasma biomarkers related to acidity of the diet to further confirm the precision of NEAP or PRAL values. Thirdly, the observational design of the current study could not conclude causality between dietary acid load and mortality rate, however, acceptable number of participants and the longitudinal design of the study could best address the association between variables. Moreover, this is the first study revealing the association between dietary acid load and cardio-metabolic risk factors and ten year survival among patients underwent CABG.

## Conclusion

This study depicts positive association between dietary acid load and 10-years survival in patients underwent CABG after adjusting for potential confounders. Our findings support the hypothesis that dietary acid load would have an important impact on several cardiovascular risk factors and reduced survival in patients underwent coronary artery bypass grafting. The results of our study suggest that maintaining an adequate acid-base balance can contribute to longevity by reducing the risk of mortality. Further randomized trials and prospective studies are needed to confirm our results.

## Supporting information

S1 Dataset (SAV)

## Abbreviation

CVD: Cardiovascular disease
CHD: coronary heart disease
CAD: coronary artery disease
NCD: non-communicable disease
DAL: Dietary acid load
PRAL: potential renal acid load
NEAP: Net endogenous acid production
CABG: Coronary artery bypass grafting
HDL: high density lipoprotein cholesterol
LDL: low density lipoprotein cholesterol
TC: total cholesterol
TLGS: Tehran Lipid and Glucose Study
DBP: diastolic blood pressure
SBP: systolic blood pressure
